# Social autopsy for identifying causes of adult mortality

**DOI:** 10.1371/journal.pone.0198172

**Published:** 2018-05-31

**Authors:** Mamta Gupta, Manmeet Kaur, P. V. M. Lakshmi, Shankar Prinja, Tarundeep Singh, Titiksha Sirari, Rajesh Kumar

**Affiliations:** School of Public Health, Post Graduate Institute of Medical Education & Research, Chandigarh, India; TNO, NETHERLANDS

## Abstract

Verbal autopsy methods have been developed to determine medical causes of deathforprioritizing disease control programs. Additional information on social causesmay facilitate designing of more appropriate prevention strategies. Use of social autopsy in investigations of causes of adult deaths has been limited. Therefore, acommunity-based study was conducted in NandpurKalour Block of Fatehgarh Sahib District in Punjab (India)for finding social causes of adult deaths. An integrated verbal and social autopsy toolwas developed and verbal autopsies of 600 adult deaths, occurring over a reference period of one year, were conducted in 2014. Quantitative analysis described the socio-demographic characteristics of the deceased, number and type of consultations from health care providers, and type of care received during illness. Qualitative data was analyzed to find out social causes of death by thematic analysis. The median duration of illness from symptom onset till death was 9 days (IQR = 1–45 days). At the onset of illness, 72 (12%) deceased utilized home remedies and 424 (70.7%)received care from a clinic/hospital, and 104 (17.3%) died withoutreceiving any care. The number of medical consultations varied from one to six (median = 2). The utilization of government health facilities and qualified allopathic doctor increased with each consultation (p value<0.05). The top five social causes of adult deaths in a rural area of Punjab in India. (1) Non availability of medical practitioner in the vicinity, (2) communication gaps between doctor and patient on regular intake of medication, (3) delayed referral by service provider, (4) poor communication with family on illness, and (5) perception of illness to be ‘mild’ by the family or care taker. To conclude, social autopsy tool should be integrated with verbal autopsy tool for identification of individual, community, and health system level factors associated with adult mortality.

## Introduction

The information on the number, causes, and determinants of mortality are essential for determining diseases of public health importance.[1]It has been widely accepted that fully functional Civil registration and vital statistics (CRVS) system is the most optimal source of mortality data. However, in two-third of the countries, majority of deaths are not registered and information on the cause of death is often unreliable. This is because in most of these countries including India, more than 50% deaths occur at home. Hence, the CRVS is not able toprovide medical cause of these deaths.[2,3] In such a situation, an alternate method of verbal autopsy has beenused in many countries like China and Indiato determine the ‘probable medical cause of death’.[4]

Cause specific mortality datahelpsin identifying diseases having high morbidity and fatality rates. Thus, prioritization of the disease can be done for which medical intervention is needed.[5,6] However, mere development of the intervention for a disease may not prevent or cure it. For instance, top ten causes of adult mortality are medically avoidable but still deaths occur due to these causes across the world.[7]Despite the availability of highly efficacious medical interventions for a particular illness, some people survive while others die. For preventing the death, timely availability and utilization of the intervention is important which itself is governed by several social, behavioural and health system factors, collectively known as social causes.[8,9]

Attempts have been made to collect and use information on social causes along with medical causes to prevent maternal, infant and child deathsin countries like Nigeria, Malawi, India and Bangladesh.[10–13]For maternal deaths, the three delays model[14]and for child deaths, pathway to survival model[15]have been adopted to explore social or behavioural factors operating at household, community and health system level. InIndia, MAPEDIR(Maternal and Perinatal Death Inquiry and Response) was piloted in ten high mortality states whichhas been scaled now to Maternal and Child Death Review at the national level.[13,16,17]However, use of these methods in assigning social and medical cause in adult deaths islimited to specified diseases only.[18–20]

With the realization of the fact that the utility of information on medical cause of death is incomplete without its social causes, an approach of determining the social causes termed as ‘social autopsy’ gained impetus.[8,9]This led to the development of integrated verbal and social autopsy (VA&SA) tool where a social autopsy tool is developed and then integrated with any existing verbal autopsy tool. Hence, present study was conducted to develop and utilize a social autopsy tool for finding social causes of adult deathssuch as socio-economic status, behaviors, and health system factors.

## Methods

### Ethical considerations

The ethical approval was provided for the PhD thesis titled, “Performance of verbal autopsy methods in identifying causes of adult mortality” by the Institutional Ethics Committee of Post Graduate Institute of Medical Education and Research, Chandigarh, India. The current paper is the outcome of an objective of the thesis.

A written informed consent was obtained from the study participants based on the ethical guidelines. Respondents were allowed to withdraw from the study at any time. Confidentiality was ensured. Health related information was provided to the family members of the deceased on the risk factors of major causes of death among adults, and about the availability of health facilities including ambulance services in their vicinity.

### Study setting

This cross-sectional observational study was conducted in Punjab, a North Indian state where 80% of the deaths occur at home.[21]Hence, Community Development Block NandpurKalour of District Fatehgarh Sahib of Punjab state was purposively chosen for this study. The population of this block was 221,737, of which 59% people reside in rural areas in 172 villages which are catered by 25 sub-health centers.[22]

### Study population

Using an integrated mixed methods research approach, verbal and social autopsies of six hundred adult deaths (aged 20years and above)were conducted that occurred amongresidents in the study villages over a reference period of one year. The sample size calculation was based on the primary objectives to assess the performance of various verbal autopsy methods described elsewhere.[23]

### Study tools

Two tools were used to collect data on social causes. Both tools were semi-structured. The verbal autopsy tool was based on adult verbal autopsy tool developed by Kumar *et al*[5] and was added to asemi- structured social autopsy (SA) tool to develop integrated verbal and social autopsy tool. For developing SA questionnaire, already existing questionnaires used for conducting maternal and child death audits were reviewed as no social autopsy tool for adults was available in the literature. The verbal and social autopsy tool from Himachal Pradesh was used as a base to develop the social autopsy tool for adults.[24]

Validity of the integrated VA & SA tool was established by expert review. Reliability of the tool was established by test-retest method in 20 adult deaths. The tools were translated to Hindi and Punjabi and back-translated to English. Pre-testing was done in 60 adult deaths. Necessary changes were made after pre-testing for tool finalization.

The first part of the integrated verbal and social autopsy tool captured information on socio-demographic characteristics of the deceased along with information regarding personal habits like use of tobacco, alcohol or any other drug and about the cause of death as told by the family member(s). The Audit- C, a brief alcohol screen tool was used to identify persons who have hazardous drinking or have active alcohol use disorders.[25]The second part was an open-ended question (narrative section) to record the sequence of events, symptoms, signs and care or treatment received during the illnessin detail as described by the respondent. The third part consisted of only closed-ended questions (structured) under three sections: section one captured the past medical history of the deceased (for example if the deceased had history of any medical condition in the past like high blood pressure or diabetes or stroke etc.); section two consisted of 20 filter questions on major signs and symptoms of illness preceding just before death like history of fever or cough or abdominal pain etc., and section three comprised of 20 modules having detailed questions about the major signs and symptoms captured in section two. The fourth part of the study tool was devoted to social autopsy which consisted of both open and closed-ended questions (semi-structured) for capturing information on type of symptoms and their duration recognized by the family at the onset of illness, social factors influencing the preventive and treatment seeking behaviour of individual, family and community, pattern of health services utilization (type of health providerand place of consultation- public health facility/ private health facility), factors leading to delay in accessing health care for each consultation during the final illness and type of treatment received at the health facility. The respondents were asked an open-ended question in the end about his/her views on how that death could have been prevented to explore the social cause of death(data collection tool provided as supplementary material).

### Data collection

A list of the sub-healthcenters along with the villages covered was obtained from the Department of Health & Family Welfare, Punjab.[26]A sub-health center is a peripheral level health post in the rural areas catering to a population of about 5000 in plains and 3000 in hilly, tribal and backward areas in Indian health care system. The basic health care services in sub-health center are provided by one female health worker (Auxiliary Nurse Midwife), one male health worker (Multipurpose Health Worker) and fivevolunteerAccredited Social Health Activists.

A list of all the villages catered by each sub-health center was prepared to keep a track of whether a village has been included in the study or not. As NandpurKalour Block is the field area of the Institute, the field visits for conducting verbal autopsies were planned according to the field activities of the Institute. Thus, the required sample size of 600 VAs was obtained from 159 villageswhich were catered by 22 sub-health centers.

Before starting data collection, the researcher (MG) received online training for conducting verbal autopsy. The online training consisted of the training modules for conducting verbal autopsies along with the exercises to differentiate good and bad verbal autopsies.[27] This was followed by a one day training workshop organized at School of Public Health, Post Graduate Institute of Medical Education and Research, Chandigarh on 23rd January 2014. Further, regular handholding on conducting verbal autopsy was done by a senior researcher who was the most experienced on verbal autopsyin the field for good quality data. Data collection was done from 1st February 2014 to 31st December 2014. Verbal autopsies of only those deaths which had occurred within past one year from the date of interview in the age group of 20 years and above were conducted for the study.

Key informants of the village (community health workers like Accredited Social Health Activist, Auxiliary Nurse Midwifery, Anganwadi Workers; Village Head, School Teachers etc.) provided the information on deaths. They also helped in approaching the person who was with the deceased during the last stage of life. A total of 602 households were approached for the interview. VAinterviews could not be done in two houses because of migration of the family members after the death of the deceased. The interview was conducted with the caretaker who was with the deceased before death. After explaining the purpose of the study to the respondents, data was collected using integrated verbal and social autopsy tool. The average duration of interview was 30–45 minutes. Social autopsy data was collected for every consultation made by the deceased during his/her final illness, thereby, in many cases; multiple record sheets had to be used (fourth part of integrated verbal and social autopsy tool).

Five community physicians received online training for assigning medical causes of death using ICD-10 classification.[28] The data collected were presented to two trained community physicians who assigned a medical cause of death using the VA questionnaire. Qualitative and quantitative analysis of social autopsy data was done to explore the social causes of death as reported by the family members of the deceased.

### Data analysis

SPSS-16 software was used for data entry and quantitative analysis. Univariate and bivariate analysis was done to describe type of care received during the illness by the socio-demographic characteristics of deceased, and by the number and type of consultations from various types of health care providers. Statistically significant association of socio-demographic characteristics with type of care received by the deceased was evaluated by using chi-square test.

Thematic analysis of qualitative data was used to find out social causes leading to death as reported by the family members. At the outset, 240 codes of social causes emerged. These codes were pooled in 90 meaningful categories which were further merged into 60 sub-themes. Finally, 34 themes evolved as social causes of adult deaths. Linkage of these themes and sub-themes were explored using three models: ‘Pathway to Survival Model’,[15] ‘Three Delay Model’[14] and ‘Social-Ecological Model’[29]

Following operational definitions were used in the social autopsy analysis.

Health system: It included both government and private health facilities; types of providers included are both formal and informal health providers. Traditional treatment was conceptualized as per WHO definition which refers to health practices, approaches, knowledge and beliefs incorporating plant, animal and mineral based medicines, spiritual therapies, manual techniques and exercises, applied singularly or in combination to treat, diagnose and prevent illnesses or maintain well-being.[30]Time lapse of more than four hours between recognition of illness and receiving health care was termed as delay for the ‘Three Delay Model’.

## Results

The results are presented in three sub- sectionsto explain the linkage among social causes using three models;after describing the the socio-demographic characteristics of the deceased., First sub-section describes the findings as per ‘pathway to survival model’. Second sub-section presents the findings of ‘three delay model’. The last sub-section deals with the application of ‘social ecological model’.

### Socio-demographic characteristics

Out of 600 adult deaths in NandpurKalour block of Punjab, 40.5% were in the age group of 61–80 years followed by 26.5% in the age group of 41–60 years. The median age of the deceased was 67 years (IQR = 50 to 80 years). There were 56.8% males and 43.2% females. Fifty eightpercentbelonged to general caste, 33% were scheduled caste or scheduled tribe and rest 9% were in Other Backward Class (OBCs). Sixty five percent were illiterate. Cultivators constituted 15% of the sample while 11.7% were unskilled workers. Four percent were unemployed; 29.5% did not work due to old age and 26.3% were housewives (Table 1 & S1 Table).

**Table 1:** Type of care received by the deceased by socio- demographic characteristics in NandpurKalour Block, Punjab, India.

Socio-demographic characteristics		No Care Received		Home Remedy		Direct Consultation		Total		p
		N = 104		N = 72		N = 424		N = 600		value
		n	%	N	%	N	%	n	%	
Age	20–40	31	38.3	5	6.2	45	55.6	81	13.5	**<0.05**
	41–60	24	15.1	15	9.4	120	75.5	159	26.5	
	61–80	28	11.5	33	13.6	182	74.9	243	40.5	
	80+	21	17.9	19	16.2	77	65.8	117	19.5	
Gender	Female	34	13.1	30	11.6	195	75.3	259	43.2	**<0.05**
	Male	70	20.5	42	12.3	229	67.2	341	56.8	
Caste	Scheduled Caste/ Scheduled Tribe	31	15.7	21	10.6	146	73.7	198	33.0	0.8
	Other Backward Class	9	16.7	7	13	38	70.4	54	9.0	
	General Caste	64	18.4	44	12.6	240	69	348	58.0	
Marital status	Never Married	18	36	2	4	30	60	50	8.3	**<0.05**
	Married	48	14.6	47	14.3	233	71	328	54.7	
	Widowed/ Divorced/Separated	38	17.1	23	10.4	161	72.5	222	37.0	
Education	Illiterate	54	13.8	56	14.3	281	71.9	391	65.2	**<0.05**
	Primary & literate with non-formal education	11	16.7	6	9.1	49	74.2	66	11.0	
	Upto senior secondary	37	28.5	9	6.9	84	64.6	130	21.7	
	Diploma/ Graduate/ Postgraduate	2	15.4	1	7.7	10	76.9	13	2.2	
Occupation	Non- workers (Unemployed, old, students)	36	16.7	28	13	151	70.2	215	35.8	0.08
	Unskilled & skilled Worker	26	29.9	9	10.3	52	59.8	87	14.5	
	Cultivator/ Own business	14	13.3	13	12.4	78	74.3	105	17.5	
	Service/ Retired from service	7	20	2	5.7	26	74.3	35	5.8	
	Housewife	21	13.3	20	12.7	117	74.1	158	26.3	

Out of the 600 deceased, 175 (29.1%) consumed alcohol. Among those who consumed alcohol, 122 (69.7%) were identified as having hazardous drinking or active alcohol use disorders as per Audit-C score.[25] Tobacco smoking was reported among 49 (8.2%) while 71 (11.8%) were reported to be tobacco chewers. Sixteen 16 (2.7%) were drug users. All the substance users (alcohol/ tobacco/ drugs) were males.

According to the respondents, top five causes of death were: ‘natural death’ in old age 125 (21%), ‘attack’ 96 (16%), cancer 51 (8.5%), heart attack 46 (7.7%) and accidents 34 (5.7%). The term ‘attack or bulla’ was used by respondents mostly for stroke as well as where they were not clear whether it was a heart attack or a stroke. The age and gender-wise distribution of causes of death reported by respondents are presented in S2 and S3 Tables.

### Care pathways

**Fig 1:**
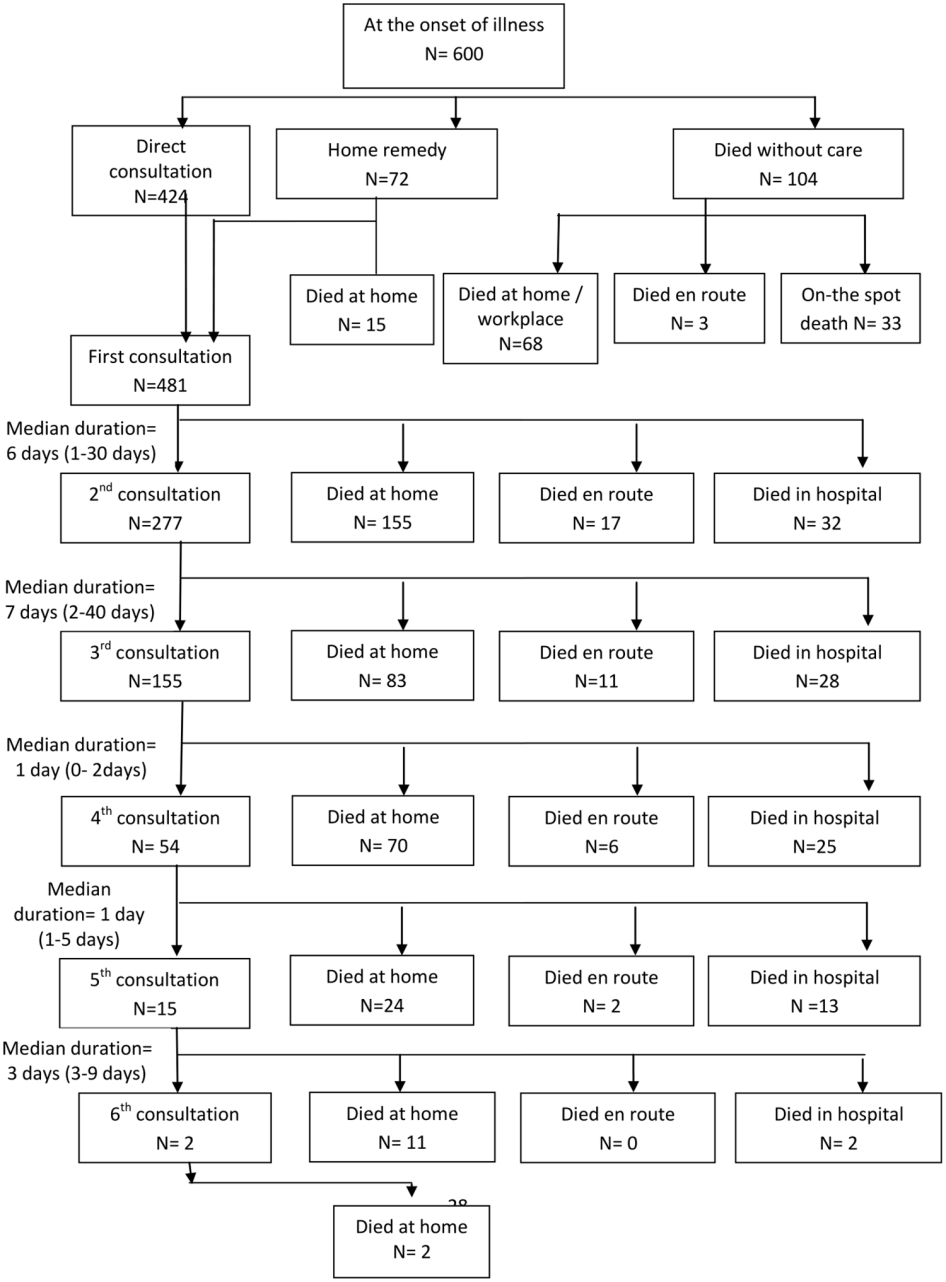
Care pathway of 600 adult deaths in NandpurKalour Block, Punjab.

At the onset of symptoms, 72 (12%) individuals resorted to home remedy, 424 (70.7%) went outside home to seek care, and 104 (17.3%) died without receiving any care **(**Fig 1). The number of consultations per individual varied from one to six (median: 2 consultations).

The type of care received by the deceased during his/ her illness was associated with socio-demographic characteristics of deceased like age, gender, marital status, and education (Table 1). For example, out of 104 individuals who did not receive any medical care before death, 67.3% were males, and 20-40-year olds constituted the highest proportion (29.8%) in this group. The main reason for not receiving any care was ‘on-the-spot’ death due to injuries or sudden ‘attack’ in younger people, whereas in older age group ‘lack of care’ and in a few cases ‘neglect’ from the family members was the main reason for not receiving care. The issue of ‘neglect’came up while interacting with the neighbours of deceased as mentioned in the following excerpt:

“*Two years back, he had given all his property to his children. After that nobody took care of him. He was on bed for long. Bed sores were there, but nobody cleaned his wounds*……*no one cared for him*.”

Few individuals did not seek care as they ignored their illness or they were either addicted to alcohol or drugs. Doctors had asked them to quit drinking/ drugs earlier but they could not quit it. Hence due to fear of getting the same advice from the doctor, they did not tell the family members about the illness and were not willing to seek medical care. One of the family members reported:

“*He was taking too much of alcohol. He had pain in abdomen a few years ago. He was advised to stop drinking. But he did not stop. He was ‘just drinking’ for a month before death. No food, no talk, and died*……”

In some cases, the family members did not notice any major symptom of illness prior to death as mentioned by one of the respondents:

“*He was not ill…was taking less food since one or two days. That day he just ate light food and died*.”

In a few cases, the family thought that the condition was not treatable like tuberculosis and mental illness. Hence, they did not seek any care or family considered that there was no use to consult the doctor as old persons do not respond to medications,

“*Bapuji (Grandfather) was very old. Medications did not work for him so we did not go to any doctor*.”

As first action following illness, 72 individuals resorted to home remedy. Majority of them were in the age group of 60 years and above. In most of these cases family members believed that symptoms like paralysis, jaundice, fits, cough and cold required traditional treatment. Moreover, they did not consider symptoms like common cold, diarrhea, fever, joint pains severe enough to seek care from doctor but relied upon home remedies like use of ‘ginger-honey-black pepper mixture’ for treating cough; ‘sugarcane juice’ for jaundice and use of ‘certain herbs’ for treating epilepsy. ‘Opium dissolved in water’ was reported to be most commonly used home remedy for paralysis- ‘attack’. Many also reported the use of self-medication such as paracetamol or crocin for fever, cetirizine for common cold, norflox in diarrhoea and abdominal discomfort, disprin for thinning blood and paracetamol in joint pains. Most of those who initially used home remedies or self-medication for few days had shifted to medical consultation subsequently.

Most (80.2%) of the deceased who received medical consultation outside their home did so due to the ‘perceived severity’ of illness. In some cases family members could not understand the nature of disease but perceived the symptoms like sudden loss of consciousness, raised anxiety, perspiration, increased heart beat etc. to be serious.

For the first medical consultation, 375 (78%) preferred private health facility and rest accessed government health facility. Among these, 291 (60.5%) had consultation with the allopathic qualified doctor and the rest resorted to non-allopathic unqualified providers. These findings were supported by the following excerpts:

“*Peeliya (jaundice) is because of some supernatural power. We give sugarcane juice along with that we take him to‘Sanaurwala baba’. He put a thread around the wrist. The knots open by themselves with the reduction of jaundice*.”

“*In case of snake bite we take the patient to temple. There the senior baba performs some ‘pooja’ and with the help of sharp object, the poison is drained out. Everybody gets saved. In case of my wife we also went there. But there was a ‘junior baba’. Perhaps he was not very well trained but he tried his best. When he failed to do so, he asked us to take her to PGI. We reached PGI at 6 a.m. she was admitted to emergency and died there at 7 a.m. Perhaps if ‘senior baba’ had been there, he could have saved her*!!”

“*If somebody gets paralytic attack we give ‘afeem’- opium by dissolving in water. The patient gets stabilized some what. The body part gets saved from paralysis. Then we take the person to ‘BoongaTibbi’ near Patiala. There two girls are blessed with the art of treating paralysis. They give two injections; one is like the color of water and other is of pinkish tone. They charge 500 rupees for it. God knows what type of injection they give; but the patient gets relieved. Not only us but everyone in our village go there in case of paralytic attack*.”

Hence, for illnesses like jaundice, snake bite and paralysis, the community preferred to avail treatment from unqualified private health provider (faith healers).

It was observed that the utilization of government health facility increased over private with each subsequent consultation (p <0.05). The consultation of allopathic qualified doctor also significantly increased with each subsequent consultation (p <0.05) (Table 2 & S4–S7 Tables).

**Table 2:** Pattern of health care utilization according to the number of medical consultations in NandpurKalour Block, Punjab, India.

Health Care Utilization		1st		2nd		3rd		4th		5^th^		6^th^	
		N = 481	%	N = 277	%	N = 155	%	N = 54	%	N = 15	%	N = 2	%
Place of consultation	Government	106	22.0	122	44.0	85	54.8	25	44.4	3	20.0	2	100
	Private	375	78.0	155	56.0	70	45.2	29	55.6	12	80.0	0	0
Type of provider	Allopathic doctor	291	60.5	234	84.5	130	83.9	46	85.2	8	53.3	2	100
	Non- allopathic provider	190	39.5	43	15.5	25	16.1	8	14.8	7	46.7	0	0
Condition of deceased at the time of consultation	Unconscious	59	12.3	28	10.1	14	9.0	10	18.5	1	6.7	0	0
	Semi- conscious	83	17.3	65	23.5	38	24.5	9	16.7	4	26.7	0	0
	Fully conscious	339	70.5	184	66.4	103	66.5	35	64.8	10	66.7	2	100
Reason to seek consultation	Satisfied with the health care	201	41.8	31	11.2	16	10.3	1	1.9	15	100	1	50.0
	Suggested by many people	125	26.0	132	47.7	62	40.0	28	51.9	11	73.3	1	50.0
	Near to home	196	40.7	27	9.7	9	5.8	2	3.7	1	6.7	0	0
	Cost effective	39	8.1	18	6.5	14	9.0	5	9.3	1	6.7	0	0
	Perceived Illness to be minor	98	20.4	0	0	0	0	0	0	0	0	0	0
	Big hospital/ competent doctor	18	3.7	67	24.1	48	31.0	18	33.33	0	0	0	0
Mode of transport	Own car/taxi	242	50.3	169	61.0	76	49.1	40	74.1	2	13.3	1	50.0
	On foot	63	13.1	9	3.2	4	2.6	0	0	6	40.0	1	50.0
	Bus	37	7.7	51	18.4	43	27.7	12	22.2	6	40.0	0	0
	Motor cycle	31	6.4	0	0	0	0	0	0	0	0	0	0
	Ambulance	17	3.5	14	5.1	3	1.9	0	0	0	0	1	50.0
	Doctor came home	90	18.7	34	12.3	29	18.7	2	3.7	1	6.7	0	0
Advice given	Outdoor Patient Department	295	61.3	103	37.2	54	34.8	11	19.4	8	53.3	0	0
	Indoor Patient Department	106	22.0	141	50.9	82	52.9	42	77.8	6	40.0	2	100
	Referral	80	13.7	33	11.9	19	12.3	1	1.9	1	6.7	0	0
Reason for referral	Provider not able to handle problem	73	91.3	22	66.7	16	84.2	0	0	1	6.7	0	0
	Medicines/Tests not available	12	15.0	17	51.5	19	100.0	1	100	1	6.7	0	0
	Equipment not available	18	22.5	15	45.5	1	5.3	0	0	1	6.7	0	0

Existence of health provider near the households was a major factor influencing the choice of healthcare provider. This observation is also supported by the data on time taken to approach the health provider and mode of transport used. The median time to approach the health provider rose from 15 minutes from first consultation to 47.5 minutes in subsequent consultations.

The use of own car or taxi and bus increased with each consultation. This also depended on the condition of the deceased at the time of care seeking. In the first consultation, 13% approached the health care provider on foot. This indicated the conscious state of the deceased at the onset of illness, whereas, more people were in the semi-conscious and unconscious state during subsequent consultations (Table 2 & S4–S7 Tables).

### Causes of delay in receiving care

The median duration from illness recognition until home care was four hours (IQR: 1–10 hours), and until care was sought from outside the home it was one day (IQR: 0–3 days). Those who initially receive home remedies, their first medical consultation from outside home were after the median delay of 3 days (IQR: 0.5–10 days). Overallmedian duration from illness recognition till death was 9 days (IQR: 1–45 days). Several factors led to the delay in receiving care.

Out of 424 direct consultations, no delay in receiving care was found among 158 (37%) cases, while among the rest 266 individuals, the reason for delay were perceived sickness to be ‘mild’ in 236 (89%) followed by unavailability of someone to accompany (4%), non-curable condition (4%), far off health facility (3%), deceased was not willing to seek care (3%), non-availability of transport (2%), lack of decision making power due to gender issues (2%) and high cost of treatment (2%).

Four types of delays were noticed. The first delay was from the onset of symptom to illness recognition by the family, and it also relates to delay in deciding whether to seek care or not in 72% cases (n = 432). In this type of delay, there was interplay of multiple factors operating at individual, family and community levels. The awareness about symptoms and their severity, age, and gender influenced the decision when to seek care as well as whether to seek care or not.

Second delay corresponded to decisions about where to seek care among 43% (n = 258). This decision was governed by factors like awareness of the family and community about the ‘appropriate’ place of treatment, perceived accessibility and affordability of the treatment. The belief in the practice of community to utilize informal treatment providers influenced the decision about the place of consultation. However, lack of awareness regarding the ‘appropriate’ place of treatment was also a major barrier which caused the delay.

Third delay involved the actual delay in approaching health facility after the decision by the family among two individuals only. It included factors such as availability of health facility at longer distance and high cost of treatment. Fourth delay was related to health system factors. Once the person had approached the health system, factors like availability of staff, equipment, medicines, diagnostics, competency of health provider, and attitude of health facility staff played a role in the final outcome of the illnessamong 75% individuals (n = 450).

### Social causes

**Fig 2:**
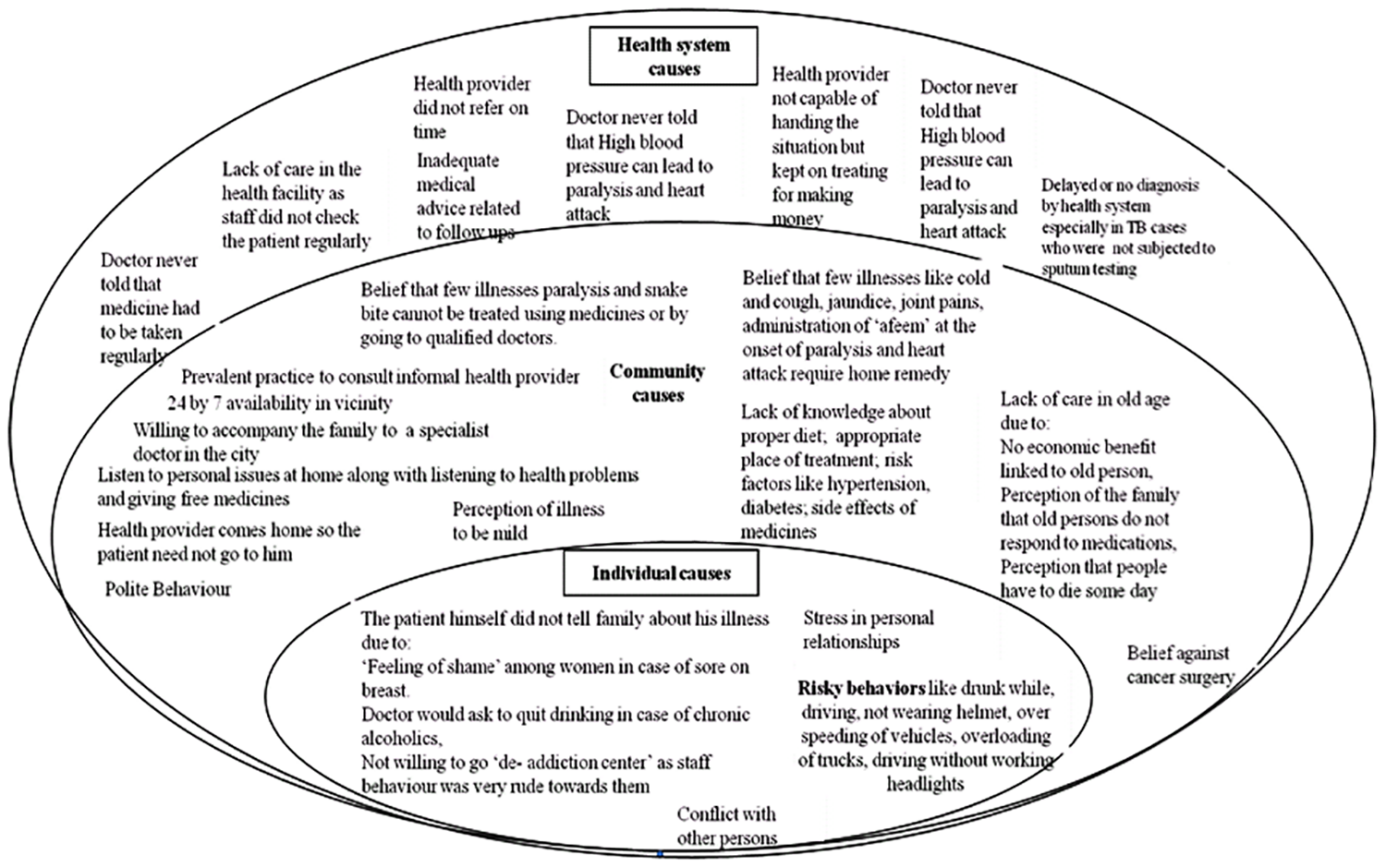
Social causes of 600 adult deaths based on Social- Ecological Model in NandpurKalour Block, Punjab, India.

The social causes of adult deaths could be broadly grouped into three categories- individual, community (including family) and health system. To understand the linkages among individual, community and health system level causes, the themes were classified as per Social-Ecological Model[29](Fig 2).

Individual level causes were: not willing to quit drinking and smoking habit, not willing to wear helmet, not willing to tell his/her family members about illness due to poor communication etc. Community level causes were: prevalent practice among people to consult informal healthcare provider or delayed recognition of symptoms by the family members either due to ignorance about the disease symptoms or the symptoms of the disease were not perceived to be serious enough to warrant their attention. The health system level causes included factors such as lack of communication between doctor and patient about regular intake of medicine to manage chronic conditions, delayed referral, and lack of capacity in managing the patients.

**Fig 3:**
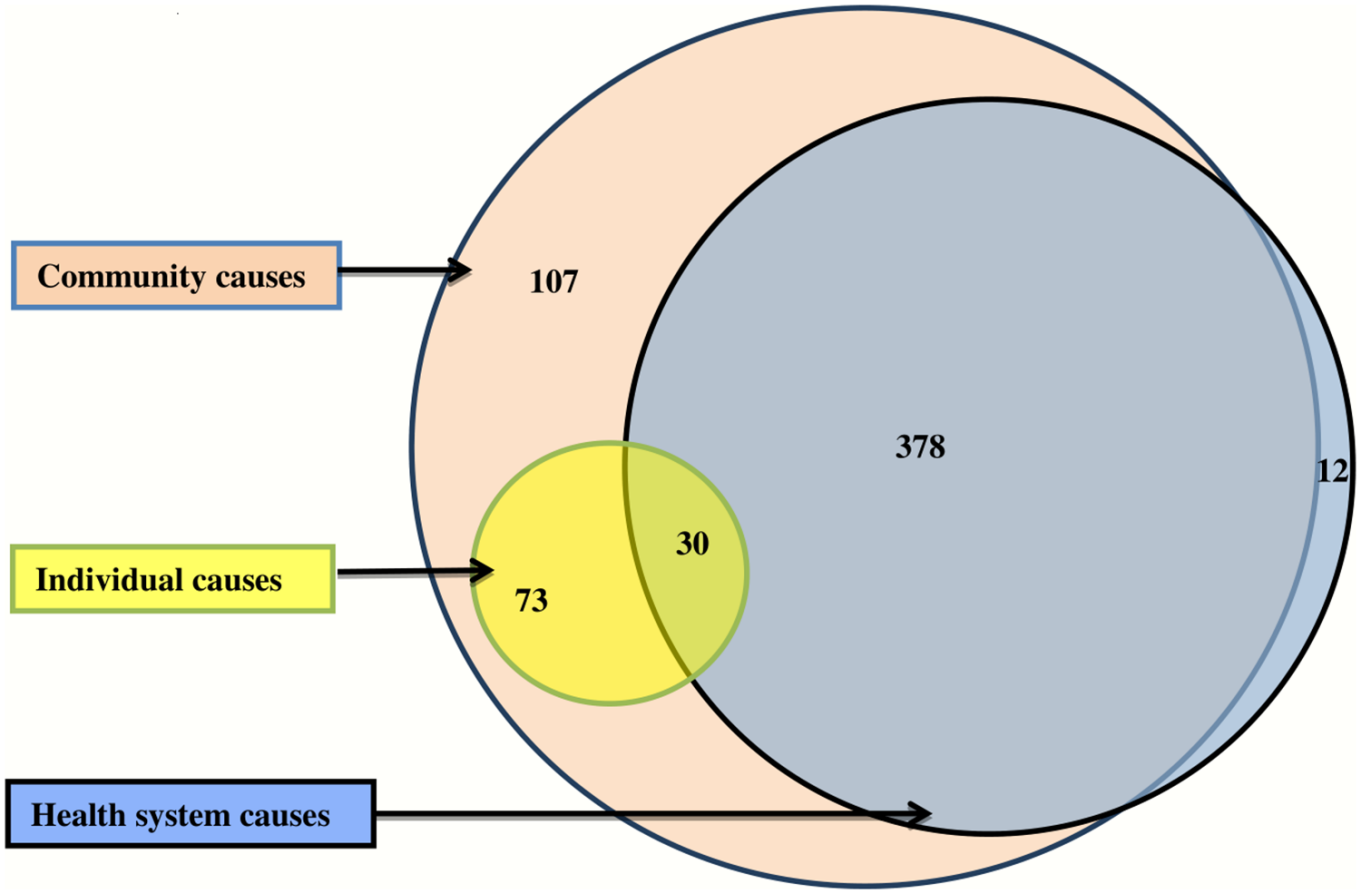
Interaction of social causes among adult deaths in NandpurKalour Block, Punjab, India.

The three types of social causes (individual, community and health system) overlapped with each other in 30 (5%) individuals (Fig 3). There was overlapping of social causes at community and health system level also in 378 (63%). As an individual is not an ‘independent unit’ and the behavior of the individual is also a reflection of the ‘community behavior’, the social causes attributed to an individual can also be considered within the community causes. Community causes alone were responsible among 108 (18%), while 12 (2%) deaths had occurred due to inadequate attention by the health system.

**Fig 4:**
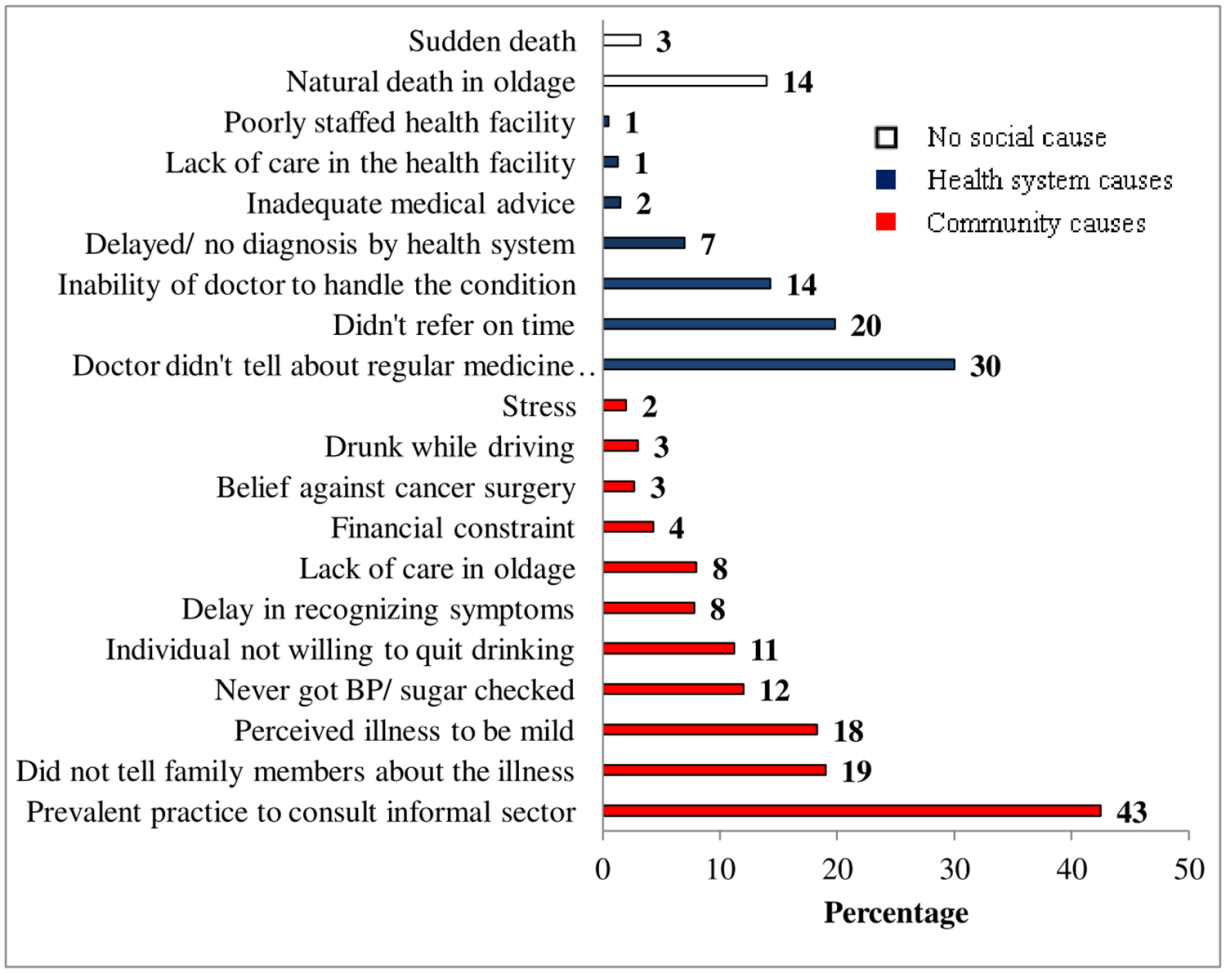
Social causes leading to adult deaths in NandpurKalour Block, Punjab, India.

Analysis of social autopsy indicated multiple social causes. The top five social causes responsible for adult deaths were: the prevalent practice in the community for consulting informal medical practitioners (43%), poor communication between doctors and patients about the importance of regular intake of medicines in chronic conditions like hypertension and diabetes etc. (30%), delay in referral by the health care providers (20%), hiding of the illness by deceased from the family members (19%), and caretaker’s perception of illness to be mild (18%) (Fig 4).

## Discussion

Causes of adult mortality are both medical and social. However, while there are quite a good number of studies available on medical causes of death, there are only a few of studies available on social causes.

### Social causes of adult mortality

The top most social cause responsible for an individual’s death was found to be “prevalent practice of the community to consult informal medical practitioner.” First possible explanation for this type of practice could be lack of awareness among community about the ‘nature’ of the disease, ‘appropriate’ place of medical consultation, and that the treatment is ‘available’ for a particular symptom or illness. It was observed that people in rural areas still think that ‘stroke or bulla’ is incurable. Second explanation could be the presence of ‘informal health care provider’ near the households, thereby, available round the clock (day and night). The informal health care provider is looked upon as a ‘family member’ by the community; thereby the trust of the community vests in him/ her.

Top second social cause relates to health system where most of the times the health care provider do not tell the patient that certain illness like hypertensionanddiabetes etc. need to be monitored and medicines have to be taken regularly.

The third social cause was delayed referral by the health provider. This practice was observed mostly among the private health providers. They kept on treating the patient until a time the illness became serious and out of their control. The health care provider did not have enough knowledge for treating that condition but for making money, he/ she kept on calling the patient for follow-ups.

The fourth social cause was related to the fact that the deceased did not tell his/ her family members about the illness. The observed reasons were lack of open communication channel among family members like in cases of ‘breast cancer’, the women did not discuss in family about the ulcer on her breast due to ‘feeling of shame’. Another reason was ‘lack of trust’ in the family that they would take the deceased for treatment in old age. In case of chronic alcoholics and drug users, the doctor had already warned the person to leave these habits. Due to fear of family and doctor, the person did not tell the family in serious condition also and died without receiving any medical care.

The fifth social cause was the perception of the illness to be ‘mild’ by the individual or family. Factors like lack of awareness about the symptoms of a disease, few cases of ‘neglect’ by the family members in old age were related to this cause.

Initially, we used social-ecological model[29,31] for social autopsy data analysis. In this model we could classify the themes of social causes into three broad categories of causes at individual, family and community, and health system levelsuch that individual causes were encompassed under the community causes which again operated within the broader domain of policy and health system. But during analysis it was found that these social cause themes are not independent of each other; but interlinked with each other (Fig 3). Also one theme is leading to another just like in case of medical causes in which the ‘underlying’ cause of death lead to train of events to immediate cause of death. This model could have been successful in explaining these causes if only underlying cause was used like ‘one social cause leading to one death’. But often there are multiple social causes which interact with each other and lead to the death of an individual. Similarly ‘three delays model’ and ‘pathway to survival model’ was also used to find out social causes.[14,15] These models were helpful in exploring the social causes but failed to establish the linkage among the social causes. Hence, the social causes from the study were categorized as causes at individual, community and health system level which was depicted as a venn diagram (Fig 3).

**Fig 5:**
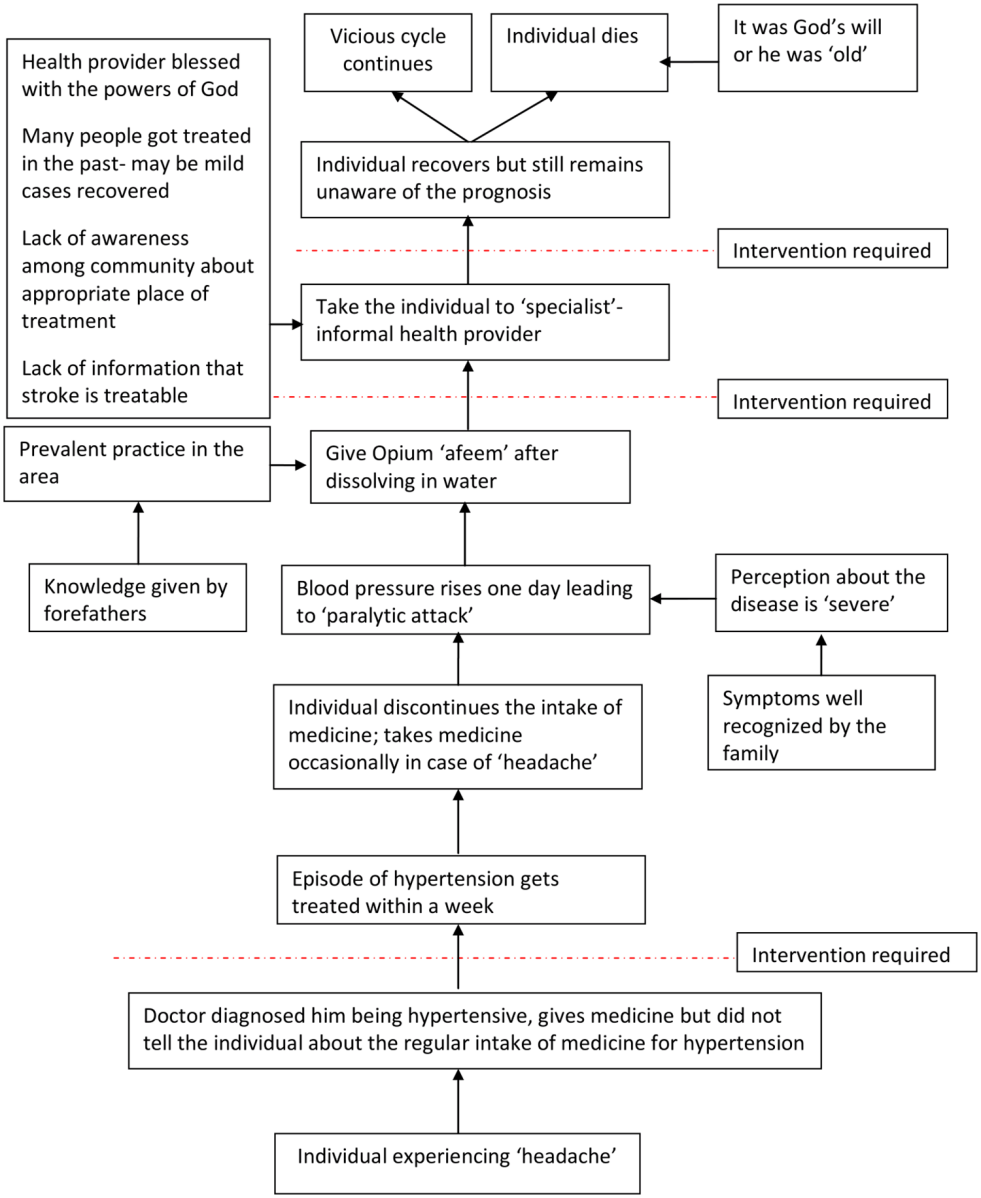
Flowchart of social causes in cases of stroke in NandpurKalour Block, Punjab, India.

The venn diagram along with the explanation for social causes support the social determinants of health model. These determinants of deaths are similar to social determinants of health[32]. For instance, in cases of stroke the chain of social events can be presented as shown in Fig 5. It can be clearly seen that the social causes are also preventable just like the medical causes of death. Researchers have advanced the concept of ‘avoidable mortality’ considering those medical causes as potentially avoidable which can be managed in the presence of timely and appropriate health care. It includes deaths which are amenable to health care (secondary prevention) as well as preventable deaths due to public health policies (primary prevention) till the age of 75 years. According to this concept, 50% deaths due to Cardiovascular Diseases are avoidable in the presence of timely health care.[7,33]

Similarly, as illustrated in a case of stroke in Fig 5, there are multiple social causes at all levels of prevention. Starting from the primordial prevention i.e., increasing the awareness among the community about the risk factors and that prevention of risk factor can lead to prevention of all those diseases which share similar risks factors. Even if the person has developed hypertension, deaths can still be prevented, if a major social cause identified in this study could be addressed i.e., inability of the health system to educate the individual, family and community about the importance of regular monitoring and medicine intake. So with the intervention of health system death could have been prevented even after occurrence of the disease. Next level of prevention is to make the community aware about the appropriate place of treatment along with some medical facts that some of the paralytic attacks or stroke episodes are self-curable and that the role of informal healthcare provider in curing these cases is negligible, this can be termed as secondary prevention. Tertiary prevention can deal with the rehabilitation of the individual which can also be done by health system by involving physiotherapists for maintain healthy life style of the individual.

Delay in deciding whether to seek care or not was a major cause in most cases, delay in choosing appropriate place of care contributed to the adult deaths in many cases, delay due to transport issues was rarely encountered, and delays related to health system causes were responsible deaths in majority of cases.

Overall, there was overlapping of multiple social causes which lead to death. The main social cause for mortality among children is the delay in deciding whether to seek care or not.[8,34] But in this study, the contribution of community factor and health system factors was approximately the same. This may be because our SA tool captured all consultations before death whereas in other studies only details of first and last consultation were taken.

### Factors associated with care pathway

The type of action at the onset of illness varied significantly with age, gender, education, and marital status (Table 1). This finding is supported by numerous studies in the literature but these studies were confined to specified causes of illness.[18–20,35]The underlying social reasons that make age and gender significantly associated with the type of care received at onset were ‘perceived severity of illness’ which further depended on presenting symptoms of illness, and other social causes like lack of care at home for elderly and women, failure to recognize illness symptoms. The distribution of causes of death varied among age groups and hence, the type of care also varied. For example, the road traffic accidents were more common among younger age groups. Hence, these “on-spot- deaths” occurred without any care. Similarly, deaths due to ‘paralysis’ and ‘heart attack’ were more common in 60–80 year age group. Hence, in this age group more people either resorted to home remedy due to lack of symptom awareness. On the other hand, with increasing age, the perception of family about the ‘illness’ of deceased become ‘mild’ justifying ‘no care is required’.

It was found that from the first consultation to subsequent consultations there was a significant increase in the usage of government health facilities and qualified allopathic doctors over private health sector and non-allopathic unqualified providers respectively (Table 2). This finding is well established in another study by Kumar and Prakash. [36] The satisfaction and faith of people on health care provider depend upon factors like easy availability round the clock, accessibility near to home or provider coming to their home. This led to health care utilization in the vicinity of home at the onset of illness. And in the vicinity, unqualified practitioners, jhaarphoonkwala baba, hakim, and vaid, flourish. Hence more utilization of non-allopathic unqualified providers was reported. Narayana (2006) has mentioned that wherever there is a government health facility, the private practitioners flourish there and normally the place is in close vicinity of the community.[37]

### Strengths of the study

To explore the social causes related to death two types of questions were used in the questionnaire; one was structured and another was unstructured narrative based. Analysis of both the questions revealed similar social causes. Hence, triangulation of data could be established. Combined VA & SA tool could be administered in 30–45 minutes to give both medical and social causes of adult mortality. Social autopsy tool used in this study could capture all the consultations sought by deceased for the illness episode before death. Thereby, it was possible to explain the pathway from survival to death in detail, which otherwise is the limitation of several social autopsy studies. This is the first comprehensive social autopsy tool and analysis model for adults for exploring major social causes of adult mortality which not only explores social factors but also interlinks them to explain social causation of death.

### Limitations of the study

This study presents multiple social causes leading to an adult death. However, to reduce adult mortality, there is a need for developing methods to identify one ‘underlying’ social cause for every death as is done for identifying one medical cause of death by using verbal autopsy data. This could provide the better actionable information to policymakers. Therefore, further analysis of verbal autopsy narratives is required to provide one social cause for every adult death. Also the VASA data may be presented to an expert panel to decide if the death could be labelled as ‘avoidable or preventable’ from both medical and social perspective.

### Conclusions

Social autopsy (SA) can be integrated with verbal autopsy (VA) to conduct comprehensive investigation of the causes of adult deaths. Integrated VA&SA tool could unravel following top five social causes of adult deaths in a rural area of Punjab in India. (1) Non availability of medical practitioner in the vicinity, (2) communication gaps between doctorand patient on regular intake of medication, (3) delayed referral by service provider, (4) poor communication with family on illness, and (5) perception of illness to be ‘mild’ by the family or care taker. Therefore, social autopsy questionnaireshould be integrated with verbal autopsy so that while designing public health programssocial factors operating at individual, community, and health system level can also be taken into consideration.

## Supporting information

S1 Table SPSS datafile consisting of socio-demographic information about deceased. (SAV)

S2 Table Sex-wise distribution of cause of death narrated by respondents in Nandpur Kalour Block, Punjab. (PDF)

S3 Table Age-wise distribution of cause of death narrated by respondents in Nandpur Kalour Block, Punjab. (PDF)

S4 Table SPSS datafile consisting of details about first consultation. (SAV)

S5 Table SPSS datafile consisting of details about second consultation. (SAV)

S6 Table SPSS datafile consisting of details about third consultation. (SAV)

S7 Table SPSS datafile consisting of details about fourth consultation. (SAV)
