# Supplementary material for: Social autopsy for identifying causes of adult mortality
Source: PLoS One. 2018 May 31;13(5):e0198172. doi: 10.1371/journal.pone.0198172 (PMC5978887; doi:10.1371/journal.pone.0198172)
Supplement: S2 Table — (PDF) [file pone.0198172.s002.pdf]

S2 Table

Sex-wise distribution of cause of death narrated by respondents in Nandpur Kalour Block, Punjab

Perceived Cause of Death	Female		Male		Total	
	N=259	%	N=341	%	N=600	%
Heart attack	58	22.3	115	33.7	173	28.8
Natural death	65	25.1	60	17.6	125	20.8
Cancer	27	10.4	24	7.0	51	8.5
Accident	4	1.5	30	8.8	34	5.7
Paralytic attack (bulla)	14	5.4	15	4.4	29	4.8
Sudden	9	3.5	17	5.0	26	4.3
Jaundice	9	3.5	15	4.4	24	4.0
Sugar	12	4.6	7	2.1	19	3.2
breathing problem	9	3.5	8	2.3	17	2.8
Kidney failure	10	3.9	5	1.5	15	2.5
Suicide	1	0.4	13	3.8	14	2.3
Drinking habit	0	0.0	12	3.5	12	2.0
TB	5	1.9	4	1.2	9	1.5
Bedridden due to long illness	5	1.9	3	0.9	8	1.3
Mental illness	4	1.5	3	0.9	7	1.2
Fever	2	0.7	4	1.2	6	1.0
High blood pressure	4	1.5	2	0.6	6	1.0
Infection in blood	2	0.7	4	1.2	6	1.0
Joint pains	4	1.5	2	0.6	6	1.0
Fall	3	1.1	2	0.6	5	0.8
Stomach problem	2	0.8	3	0.9	5	0.8
Snake bite	1	0.4	3	0.9	4	0.7
Unknown cause	3	1.2	1	0.3	4	0.7
Hepatitis	0	0.0	3	0.9	3	0.5
Murder	0	0.0	3	0.9	3	0.5
Skin problem	1	0.4	1	0.3	2	0.3
Typhoid	0	0.0	2	0.6	2	0.3
Urine problem	0	0.0	2	0.6	2	0.3
Anemia (khoon di kami)	0	0.0	1	0.3	1	0.2
Blood in vomiting	1	0.4	0	0.0	1	0.2
Convulsions (Daurey)	1	0.4	0	0.0	1	0.2
Cough with sputum	0	0.0	1	0.3	1	0.2
Dengue	1	0.4	0	0.0	1	0.2

Drug addiction	0	0.0	1	0.3	1	0.2
Operation not done properly	1	0.4	0	0.0	1	0.2
Pneumonia	0	0.0	1	0.3	1	0.2
Prostate enlargement (Gadood badhna)	1	0.4	0	0.0	1	0.2
Stones in stomach	0	0.0	1	0.3	1	0.2
Tetanus	0	0.0	1	0.3	1	0.2
