# Supplementary material for: Social autopsy for identifying causes of adult mortality
Source: PLoS One. 2018 May 31;13(5):e0198172. doi: 10.1371/journal.pone.0198172 (PMC5978887; doi:10.1371/journal.pone.0198172)
Supplement: S3 Table — (PDF) [file pone.0198172.s003.pdf]

S3 Table

Age-wise distribution of cause of death narrated by respondents in Nandpur Kalour Block, Punjab

**Perceived Cause of Death**	**20-40 years**		**41-60 years**		**61-80 years**		**More than 80 years**		**Total**	
	**N=81**	**%**	**N=159**	**%**	**N=243**	**%**	**N=117**	**%**	**N=600**	**%**
Heart attack	17	21.0	47	29.6	65	26.7	13	11.1	142	23.7
Natural death	1	1.2	3	1.9	45	18.5	76	65.0	125	20.8
Cancer	5	6.2	25	15.7	18	7.4	3	2.6	51	8.5
Accident	14	17.3	15	9.4	4	1.6	1	0.9	34	5.7
Paralytic attack (Bulla)	0	0.0	6	3.8	19	7.8	4	3.4	29	4.8
Sudden	2	2.5	12	7.5	10	4.1	2	1.7	26	4.3
Jaundice	3	3.7	4	2.5	15	6.2	2	1.7	24	4.0
Sugar	2	2.5	4	2.5	12	4.9	1	0.9	19	3.2
Breathing problem	2	2.5	4	2.5	10	4.1	1	0.9	17	2.8
Kidney failure	1	1.2	5	3.1	4	1.6	5	4.3	15	2.5
Suicide	11	13.6	3	1.9	0	0.0	0	0.0	14	2.3
Drinking habit	2	2.5	8	5.0	2	0.8	0	0.0	12	2.0
TB	2	2.5	3	1.9	2	0.8	2	1.7	9	1.5
Bedridden due to long illness	1	1.2	1	0.6	4	1.6	2	1.7	8	1.3
Mental illness	1	1.2	2	1.3	4	1.6	0	0.0	7	1.2
Fever	1	1.2	1	0.6	4	1.6	0	0.0	6	1.0
High blood pressure	3	3.7	3	1.9	0	0.0	0	0.0	6	1.0
Infection in blood	0	0.0	1	0.6	4	1.6	1	0.9	6	1.0
Joint pains	0	0.0	4	2.5	2	0.8	0	0.0	6	1.0
Fall	0	0.0	1	0.6	4	1.6	0	0.0	5	0.8
Stomach problem	3	3.7	1	0.6	1	0.4	0	0.0	5	0.8
Snake bite	2	2.5	2	1.3	0	0.0	0	0.0	4	0.7
Unknown cause	0	0.0	0	0.0	4	1.6	0	0.0	4	0.7
Hepatitis	2	2.5	1	0.6	0	0.0	0	0.0	3	0.5
Murder	3	3.7	0	0.0	0	0.0	0	0.0	3	0.5
Skin problem	0	0.0	1	0.6	1	0.4	0	0.0	2	0.3
Typhoid	1	1.2	0	0.0	1	0.4	0	0.0	2	0.3
Urine problem	0	0.0	0	0.0	0	0.0	2	1.7	2	0.3
Anemia	0	0.0	0	0.0	1	0.4	0	0.0	1	0.2
Blood in vomiting	0	0.0	1	0.6	0	0.0	0	0.0	1	0.2
Convulsions	0	0.0	0	0.0	1	0.4	0	0.0	1	0.2
Cough with sputum	0	0.0	0	0.0	0	0.0	1	0.9	1	0.2
Dengue	0	0.0	1	0.6	0	0.0	0	0.0	1	0.2
Drug addiction	1	1.2	0	0.0	0	0.0	0	0.0	1	0.2

Heart problem	0	0.0	0	0.0	1	0.4	0	0.0	1	0.2
Heat stroke	1	1.2	0	0.0	0	0.0	0	0.0	1	0.2
Operation not done properly	0	0.0	0	0.0	0	0.0	1	0.9	1	0.2
Pneumonia	0	0.0	0	0.0	1	0.4	0	0.0	1	0.2
Prostate enlargement	0	0.0	0	0.0	1	0.4	0	0.0	1	0.2
Stones in stomach	0	0.0	0	0.0	1	0.4	0	0.0	1	0.2
Tetanus	0	0.0	0	0.0	1	0.4	0	0.0	1	0.2
Thyroid	0	0.0	0	0.0	1	0.4	0	0.0	1	0.2
